# Supplementary material for: Prevalence of undernutrition and its associated factors among older adults using Mini Nutritional Assessment tool in Womberma district, West Gojjam Zone, Amhara Region, North West Ethiopia, 2020
Source: PLoS One. 2023 Feb 24;18(2):e0274557. doi: 10.1371/journal.pone.0274557 (PMC9955662; doi:10.1371/journal.pone.0274557)
Supplement: Attachment — Submitted filename: Response to Reviewer.docx [file pone.0274557.s002.docx]

**Authors’ response to editor and reviewers**

***Prevalence of undernutrition and its associated factors among older adults using Mini Nutritional Assessment tools in Womberma District, West Gojjam Zone, Amhara Region, North West Ethiopia, 2020.***

**Corresponding author**

Tsige Gebre, email address:[gebretsige2007@gmail.com](mailto:gebretsige2007@gmail.com)

**Subject: Submitting a revised version of manuscript**

**Object:**

We would like to thank the academic editor and reviewers for sharing the view and experience. The comments are very important and will improve the manuscript. The point-by-point responses for each of the comments and the revised manuscript are provided in the attached documents.

**Point by point responses**

We would like to take this opportunity to thank the reviewers and editor for sharing the view and constructive comments. The point-by-point responses for each of the comments are provided in the following pages.

| **Comments** | **Authors’ response** |
| --- | --- |
| ***Editor’s comment***   1. Your manuscript meets the PLOS ONE’S style 2. About occurrence of overlapping text 3. About data availability 4. Changes to data availability statement 5. About ethical statement | **Answer: thank you dear editor for your constructive comments!**   1. Thank you, we have prepared the manuscript according to the PLOS ONE’S style and please see the improvement of the manuscript. 2. Thank you, some overlapping texts are removed and corrected. Sources of information are also cited. Please see the improvement of the manuscript 3. Thank you, a. Data does not contain potentially sensitive information and shared as supporting information files. b. the minimal anonymzed data set are uploaded as supporting information files 4. Thank you dear editor, we have changed the data availability statement. Please see page 18 line 416 5. Thank you, the ethical statement is written only on the method section of the paper and see page 9 line 248. |
| **Reviewers’ comments:**  **Comments to the Author**   1. Is the manuscript technically sound and do the data support the conclusions? 2. Has the statistical analysis been performed appropriately and rigorously? 3. Have the authors made all data underlying the findings in their manuscript fully available? 4. Is the manuscript presented in an intelligible fashion and written in Standard English? | **Answer: thank you dear reviewers**   1. Thank you, we tried to improve the manuscript and conclusion sections based on the finding of the report and scientific data & please see the improvement of the manuscript and conclusion section. 2. Thank you dear reviewers, the statistical analysis have been conducted appropriately and rigorously, see regression table. 3. Thank you, the summary statistics are well written on the result section. Additionally, we have shared data publicly as supporting information files. 4. Thank you, we tried to present the manuscript in intelligible fashion and Standard English. Please see the improvement of the manuscript. |
| **Review Comments to the Author** Reviewer**#1**   1. About the rational of conducting this study 2. About adding secondary objective to the study (correlates of malnutrition?) 3. Justification on the sample size calculations 4. Graphical representation of the sampling method 5. Further justification of the statistical analysis(selection of variables (p value <0.25 ?), the actual multivariate model used, the predictor variable selection (possible correlates), the goodness of fit estimate, Multicollinearity and VIF analyses 6. About study flow diagram 7. About analysis of the association between chronic conditions and nutritional status 8. Revision of the discussion part 9. Why was only verbal consent obtained? 10. Was anemia assessed and if so a detailed discussion on its association with malnutrition may be added. 11. About revision of the recommendations 12. Adding the strengths and limitations of the study in the discussion section 13. About the correction of terminology, additional references on chronic condition and malnutrition, about utility of Figure 2, discussion on the prevalence of depression, spelling error and operational definition of physical problems | **Thank you dear reviewer**   1. Thank you, the rational of conducting this study is well addressed on the manuscript. Please see page 4 line 98-111 2. Thank you, the secondary objective of this study is factors associated with undernutrition and written on study objective (on abstraction section) and result section. 3. Thank you, initially the calculated sample size was 270 by considering p(prevalence of undernutrition among old age people from previous study)= 22.7% and d(margin of error)=5%. Then by taking into account design effect of 2 and 10% non-response rate the final sample size required of this study was 594 and see page 6 line 142-145. The non-response rate was Zero in this study because the data collectors were well trained and they visited the selected older adults’ household three times. 4. Thank you, we have showed the graphical representation of the sampling method/procedure. Please see figure 1 5. Thank you, Hosmer and Lemeshow applied statistics recommend that in Bivariable analysis variables with p value<0.25 is a candidate for multivariable analysis. In multivariable analysis variables with p value < 0.05 are declared to be significantly associated with the outcome variable. In our study, we used Hosmer and Lemeshow test of goodness of fit model. To say the model is fit p value >0.5. In our case p value is 0.734. We have checked for Multicollinearity and the existence of outliers between independent variables so there are no Multicollinearity effects between variables. The finding of VIF was 1.045 and tolerance test was 0.957 6. Thank you, we have tried to show the study flow diagram and please see figure 2 7. Thank you, we analyzed the association between chronic conditions and malnutrition. Among respondents, 146 (24.6%) had a history of chronic diseases including hypertension (8.2 %), arthritis (joint pain) (6.9%), asthma (4.7%), visual problem (4.4%), physical problems (2.5%), diabetes (0.5%) and known hemorrhoids (3.2%). In our analysis, only hemorrhoids that significantly associated with malnutrition and please see regression table(Table 3) 8. Thank you, we tried to edit and re write the discussion part of the manuscript based on received comments please see page 15 line 336-414 9. Thank you, this was wrongly written. We had prepared written consent. For those who can read and write, the data collectors gave them the consent to read and put their signature prior to data collection whereas for those who couldn’t read and write, the data collectors read the consent and provide them the consent to put their signature. Please see page 8 line 209-214 10. Thank you, since this is a population based study, we couldn’t assess anemia by asking the participants without any evidence (laboratory result). Additionally, we couldn’t afford the laboratory equipment because this study had no funding body. 11. Thank you, the recommendation part is revised and corrected please see page 18 line 429-436 12. Thank you, the strength and limitation of the study have been added in the discussion section please see page 18 line 415-419 13. Thank you dear reviewer, we tried to address these all issues please see the improvement of the manuscript. |
| **Reviewer *#2***  **Major comments**   1. Regarding the conclusions 2. Justification on why existing surveys such as DHS do not undertake nutrition surveys in this vulnerable population 3. About recommendations for the policy makers incorporating elderly population nutrition within the DHS surveys 4. Adjustment of the statistical analysis for the cluster design 5. Justification for why the authors didn’t use BMI as an indicator for assessing nutritional deficiencies in older adults   **Minor comments**  **Abstract**   1. About rephrasing the word Kebeles 2. About correcting the word “Statistical” 3. Rephrasing the last line of conclusions   **Paper**   1. Rephrasing the last line of paragraph 4 of the introduction section 2. Mentioning the anthropometric measurements 3. How many had arm span measurements and heights been measured need to be reported? 4. Reference for goodness of fit model with a p-value of 0.734 5. Details presentation on weight, arm span, height and BMI 6. Discussing limitations and strengths of the study on the discussion part 7. Incorporating major comments in conclusions 8. Formatting the references properly | **Thank you dear reviewer,**  **Answer for major comments**   1. Thank you, the conclusion section is re written please see page 18 line 425-429 2. Thank you, DHS in Ethiopia focus on the nutrition status of mothers, children and adults aged 15 to 49 (using BMI). But it doesn’t incorporate the nutritional status of these vulnerable groups of population. 3. Thank you, we have recommended the policy makers about incorporating the older adults’ nutrition within the DHS survey. Please see page 19 line 429 4. Thank you dear reviewer, there is no significance difference of older adults’ undernutrition across the clusters since the p value=0.6 5. Thank you, the authors used BMI as one part in MNA tool and mentioned under anthropometric measurements. Please see page 9 line 246   **Answer for minor comments**  **Thank you dear reviewer,**  **Abstract**   1. Thank you, the word “kebeles” is rephrased as “Village”. Please see page 2 line 38 2. Thank you, we have corrected the word “statistical” as P value less than 0.05 and see page 2 line 42 3. Thank you , we have corrected the last line of the conclusion and see page 2 line 54   **Paper**   1. Thank you dear reviewer, we have removed the sentence since it is not important. 2. Thank you, we measured weight of the participants using a digital scale SECA model 874(SECA GmbH & Co. KG, Hamburg, Germany). Please see page 8 line 217 3. Thank you, we measured the arm span and height twice per individual and reported the average. 4. Thank you, we have cited the reference and please see 10 line 274 5. Thank you, we have addressed these issues on the result section and please see page 12 line 299 6. Thank you, the strength and limitation of the study have been added in the discussion section please see page 18 line 415-419 7. Thank you, We have incorporated major comments in the conclusion and see page 18 line 424 8. Thank you, the references are properly formatted and see the improvement of the references. |
| 6. Do you want your identity to be public for this peer review?   Answer: Yes |  |
